# Ageing and Neurodegeneration: A Study of Plasma Biomarkers and Neuroimage in a Brazilian Cohort

**DOI:** 10.1002/alz70856_102923

**Published:** 2025-12-25

**Authors:** Debora Afonso Silva Rocha, Luis E. Santos, Thaís Lopes Pinheiro, Fernanda Hansen, Ivanei Bramati, Felipe K. Sudo, Paulo E. Mattos, Andrea Silveira Souza, Fernanda Tovar‐Moll, Fernanda Guarino De Felice

**Affiliations:** ^1^ D'Or Institute for Research and Education, Rio de Janeiro, RJ, Brazil; ^2^ D`Or Institute of Research and Education, Rio de Janeiro, Brazil; ^3^ D'Or Institute for Research and Education (IDOR), Rio de Janeiro, RJ, Brazil; ^4^ D`Or Institute of Research and Education, Rio de Janeiro, RJ, Brazil; ^5^ Queen's University, Kingston, ON, Canada

## Abstract

**Background:**

Plasma biomarkers have emerged as promising tools for diagnosing, prognosis, and monitoring neurodegenerative diseases. Neurofilament light chain (NfL) is a sensitive biomarker of neuroaxonal damage, associated with neurodegeneration and loss of brain volume. While studies from Europe and the United States have already defined robust reference values for plasma NfL in their local populations, the developing world lags behind. Local reference values for plasma NfL have not yet been established in Brazil or Latin America, and testing of the clinical utility of NfL is still needed. Our study aimed to contribute to the establishment of local reference values for plasma NfL and other neurodegeneration biomarkers in Brazil, by analyzing a cohort of healthy older Brazilians. Longitudinal data and samples allowed for assessing plasma biomarkers in normal aging and investigating their correlation with cortical folding.

**Method:**

This study involved samples from 77 cognitively healthy older subjects (first MRI: 70.2 ± 5 years [*n* = 77]; second MRI: 72.8 ± 6.1 years [*n* = 16], third MRI: 76.9 ± 3.6 years [*n* = 12]), enrolled in a longitudinal study on dementia at IDOR, run continuously since 2011. Structural T1w MRI (3T Philips Achieva) data were used. Global morphological measurements of the surfaces were generated with FreeSurfer v7.2.0. Plasma biomarkers were measured on a SIMOA HD‐X instrument, using commercial kits.

**Result:**

Data from our cohort allowed us to determine normal values and the annual rate of increase in plasma NfL in aging Brazilian subjects (Pearson *r* =0.4993, *p* <0.001). Correlations to with Grey matter volume (Pearson r = ‐0.29, *p* < 0.0001), White matter volume (Pearson r = ‐0.21, *p* =  0.0022), Mean Cortical Thickness (Pearson r = ‐0.22, *p* < 0.001) and hippocampus volume (Pearson r = ‐0.39, *p* < 0.0001) were also measured.

**Conclusion:**

Plasma NfL is a promising biomarker for neurodegenerative changes, showing strong correlations with aging and brain volume reductions in gray matter, white matter, hippocampus, and cortical thickness. Establishing reference values for the Brazilian population is essential for clinical application, supporting its use in diagnosing and monitoring neurodegenerative diseases.

